# Supplementary material for: Predicting visual function by interpreting a neuronal wiring diagram
Source: Nature. 2024 Oct 2;634(8032):113–23. doi: 10.1038/s41586-024-07953-5 (PMC11446822; doi:10.1038/s41586-024-07953-5)
Supplement: Supplementary file 6 — CRF and ERF predictions for individual TmY4 and TmY9 cells. Analogous to Supplementary Data 3, but for TmY target types. Shown are the top four monosynaptic pathways, the strongest pathway passing through each of the top ten intermediary types (ranking from Extended Data Fig. 7), and the trisynaptic pathway Tm1–TmY–Dm3–TmY (see the section entitled Prediction of spatial normalization). [file 41586_2024_7953_MOESM6_ESM.zip › DataS4/TmY9qΓèÑ/720575940617105595.pdf]

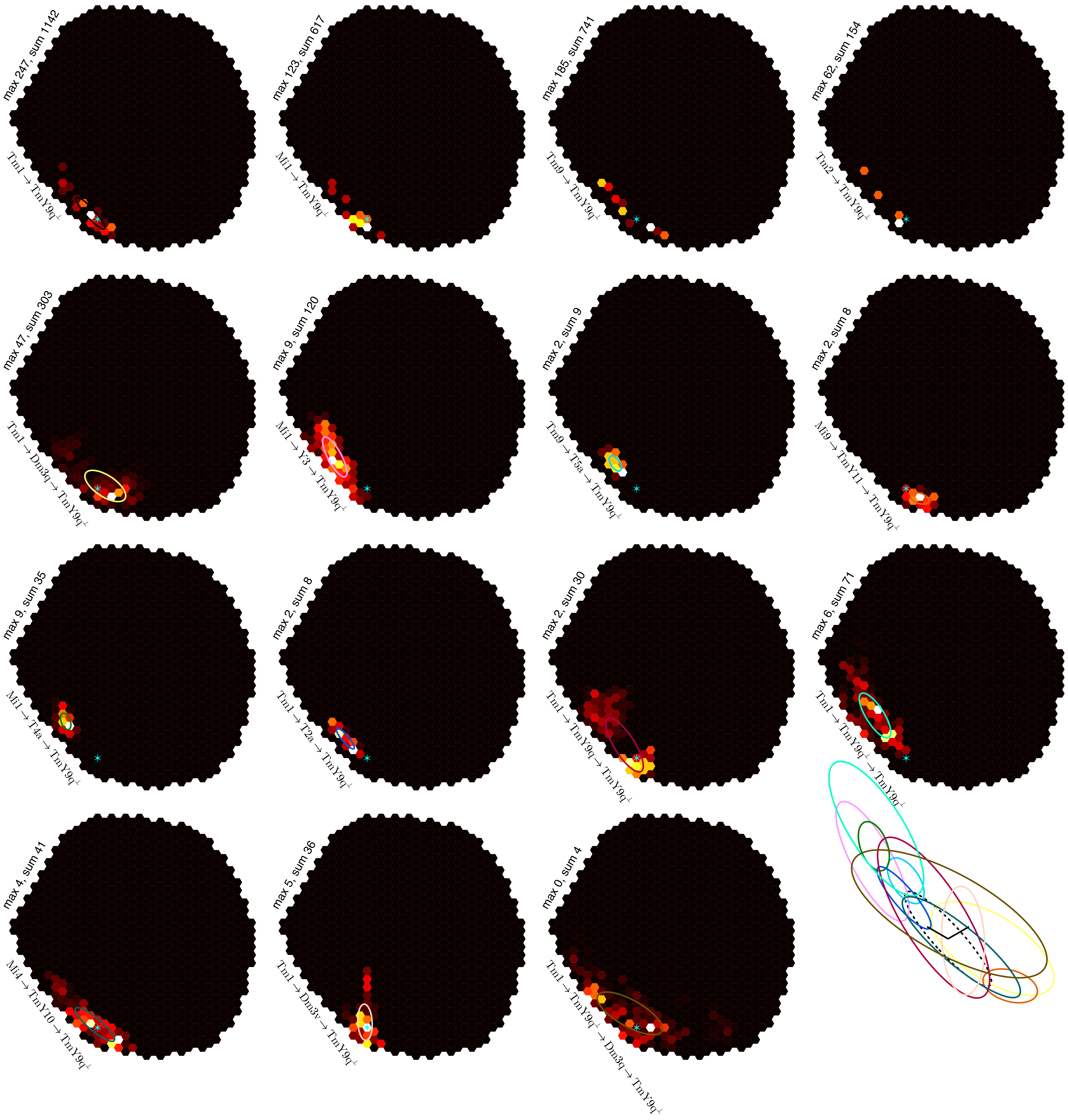
max 247, sum 1142
Tm1 → TmY9q⊥
max 123, sum 617
Mi1 → TmY9q⊥
max 185, sum 741
Tm9 → TmY9q⊥
max 62, sum 154
Tm2 → TmY9q⊥
max 47, sum 303
Tm1 → Dm3q → TmY9q⊥
max 9, sum 120
Mi1 → Y3 → TmY9q⊥
max 2, sum 9
Tm9 → T5a → TmY9q⊥
max 2, sum 8
Mi9 → TmY11 → TmY9q⊥
max 9, sum 35
Mi1 → T4a → TmY9q⊥
max 2, sum 8
Tm1 → T2a → TmY9q⊥
max 2, sum 30
Tm1 → TmY9q → TmY9q⊥
max 6, sum 71
Tm1 → TmY9q⊥ → TmY9q⊥
max 4, sum 41
Mi4 → TmY10 → TmY9q⊥
max 5, sum 36
Tm1 → Dm3v → TmY9q⊥
max 0, sum 4
Tm1 → TmY9q → Dm3q → TmY9q⊥
